# Arresting HIV: Fostering Partnerships between Sex Workers and Police to Reduce HIV Risk and Promote Professionalization within Policing Institutions: A Realist Review

**DOI:** 10.1371/journal.pone.0134900

**Published:** 2015-10-21

**Authors:** Brigitte Tenni, Jenae Carpenter, Nicholas Thomson

**Affiliations:** 1 The Nossal Institute for Global Health, Level 4, Alan Gilbert Building, 161 Barry St, The University of Melbourne, Carlton, Vic. 3010, Australia; 2 Johns Hopkins Bloomberg School of Public Health, 615 N Wolfe St, #5041, Baltimore, Maryland, 21205, United States of America; Public Health Agency of Barcelona, SPAIN

## Abstract

In many countries around the world sex work is criminalised and its regulatory control is therefore often in the hands of the police. In addition to the impact of this criminalised legal environment, much literature describes the negative impact that certain police practices can have on the ability of sex workers and the programs that work with sex workers to access essential HIV prevention, treatment, care and support services. This situation has resulted in persistent concentrated HIV epidemics among sex workers in many countries of the world. The need for multi-sector partnerships between police and HIV programs is increasingly recognised in various UN declarations and resolutions yet descriptions of the process or key ingredients required to actually establish and sustain these necessary partnerships between police and sex workers [or the programs that provide essential services to sex workers] are sparse. The paper seeks to establish key considerations and critical processes that are required to foster partnerships that if further investigated and scaled up, could result in an enhanced enabling environment for the provision of essential HIV services for sex workers around the globe. This paper is based on a realist review that investigated isolated examples of partnership formation between law enforcement and HIV programs working with sex workers. This methodology research is designed to work with complex social interventions and is based on the emerging 'realist' approach to evaluation. A realist review methodology was chosen given the paucity of relevant literature in this vein and the authors’ familiarity with the grey literature and relationships with experts who work in this sphere. The review found that political and police leadership, civil society strengthening and police reform in relation to HIV, are critical factors and key ingredients in changing the enabling environment in which sex work takes place to ensure that HIV prevention, individual and public health as well as HIV prevention and the promotion of human rights are the number one priority. Further research into this relationship is needed to provide evidence for effective HIV programming with police.

## Introduction

In many countries the buying and selling of sex is illegal and criminal law is enacted to control the industry. Regulatory control of the industry is therefore often in the hands of police. However, even where sex work is legal, street-based sex work is often conducted outside of the law. This situation makes it difficult to provide essential services to sex workers and ensures that in many countries concentrated epidemics of HIV among sex workers persist. Decriminalisation is widely regarded as the evidence-based model of sex industry regulation that best supports effective health promotion, public health outcomes, the human rights of sex workers and is the best practice model for the prevention of HIV and STIs.[1] [2] [3] [4] [5]. The decriminalisation of sex work, particularly legalising the possession of condoms and the provision of managed sex work zones facilitate more effective HIV prevention programs.[6] [7] [8] [9]

Decriminalisation is also espoused by WHO, UNAIDS, UNFPA, UNDP, sex worker organisations and human rights groups.[10][11] Despite this, all countries in the Asia and the Pacific region criminalize sex work or specific activities associated with sex work, except New Zealand and the Australian state of New South Wales.[12] Sex work is also criminalised in a host of other countries. In many of these criminalised settings, police are often perpetrators of violence against sex workers, using criminalisation to extract bribes or free sexual services [13] or targeting sex workers for arrest to fill arrest quotas. [14] People who sell sex often do not receive equal protection from the police and judiciary, and their complaints may not be taken seriously. Providing essential services to sex workers can therefore be severely compromised in environments where criminalization exists.

Given the prevalence of criminalisation, there is a growing political realisation of the need to promote partnerships between sex workers and police is also encouraged by various UN political declarations including UNESCAP Resolutions 66/10 and 67/9 [15] yet there exists only sparse descriptions of how the environment for partnerships can be fostered. While there is a significant amount of literature that describes the barriers that some law enforcement practices can create for sex workers’ health and wellbeing, there is little documentation of law enforcement practices that can actually support HIV programming, even in criminalised environments. There are however emerging examples from around the globe of sex worker programs working with police to create a greater understanding of the sex industry within police ranks and thereby aim to ensure greater access to services and greater wellbeing for sex workers.

The aim of this review is to identify and highlight examples of positive police partnerships that work with sex workers and sex worker organisations to prevent HIV transmission and to examine the aspects of these partnerships that contribute to success.

## Methodology

To best source and analyse emerging examples of partnerships between sex workers and police a realistic review methodology was chosen. This qualitative approach was chosen for its aptness for in depth analysis of a small number of cases and its flexibility with regard to sampling and is a methodology designed to work with complex social interventions based on the emerging 'realist' approach to evaluation. It provides an explanatory analysis to explore what works, when and how. A realist review does not seek to find simple solutions to complex issues but can provide a more in depth understanding of how an intervention can be made more effective. [16] A small research team from the Nossal Institute for Global Health, University of Melbourne undertook a preliminary literature review that examined the development of partnerships between law enforcement and HIV programs working with Key Populations, including sex workers. The authors first reviewed the impacts of negative policing practices on sex workers’ right to health and human rights protection to provide relevant and comparative background information before looking for positive examples of partnership formation. In keeping with realist review sampling strategies and given the paucity of peer-reviewed examples of positive partnership development between police and sex workers, the authors undertook a review of grey literature derived from leads and email communications with people familiar with programs that had sought to work with police and attempted to build studies that unpacked what specifically appeared to have assisted with partnership development. The authors were able to make use of literature collected and compiled over time and to draw on relationships fostered through working in this sphere for many years. Literature that described positive relationships between police and HIV programming in relation to sex work was included in the analysis. Due to the uniqueness of the subject matter the authors found no peer reviewed articles that described this relationship and therefore only grey literature was included in the analysis. The authors then described case examples where sex worker networks and programs working with sex workers have forged partnerships with police that have resulted in positive policing strategies and enhanced environment for access to HIV prevention services. Through an examination of these positive examples of collaboration, this paper aims to highlight key factors that need to be considered to foster and promote successful police/sex work partnerships.

## Results

### Negative policing practices towards sex workers and the implications for HIV

There is a significant amount of documentation that describes the barriers that various law enforcement cultures and practices can create for sex workers’ health and wellbeing, in particular the barriers they create for HIV prevention programs. These barriers are often compounded by the inherent legal environment in which sex work takes place but are also often the result of law enforcement cultures and practices that operate outside of legislated paradigms.

### Threats of arrest and bribery

The use of the threat of arrest and bribery by the police towards sex workers has been documented widely.[17][18][19][20] Research from Madagascar highlighted that the vast majority of interactions between police and sex workers had no legal basis. Most interactions constituted police officers attempting to gain money or sex by virtue of their uniform, their weapons or by threatening to use force. In this setting police officers represented an authority to be obeyed rather than one that has defined legal roles and responsibilities to protect them.[21] Studies from Russia have documented the widespread police practice of extracting bribes from sex workers to avoid arrest.[22][23][24] A recent report by Swaziland Action Group Against Abuse (SWAAGA) stated that sex workers were forced by police to comply with demands for free sex or sex in exchange for not being arrested. The report found that 27% of the sex workers had been arrested by state police for loitering and that 60% of those arrested were sexually and physically abused by the police.[25]

### Physical intimidation and sexual assault

In a 2009 survey of sex workers in Central and Eastern Europe and Central Asia found that 42% of sex workers reported physical abuse by police, and 37% of sex workers reported having been assaulted sexually by police. The highest rates of abuse were found in Macedonia, Ukraine, and Kyrgyzstan.[26] In Kazakhstan, police routinely arrest and beat sex workers and often force them to bribe arresting officers with money or sexual services.[27] A study in Serbia reported that police posed the greatest risk of physical violence to sex workers. Accounts from sex workers included physical violence, either threatened or enacted, in order to obtain free sexual services, arbitrary arrests, enforced admissions, and beatings by police. Alarmingly there was a common perception that the police *had the right* to beat them.[28]

A Human Rights Watch report found that in Cambodia, women and girls involved in sex work face beatings, rape, sexual harassment, extortion, arbitrary arrest and detention, forced labour, and other cruel and degrading treatment at the hands of law enforcement including officers working in the “Rehabilitation” centres run by the Ministry of Social Affairs, Veterans, and Youth Rehabilitation.[29]

In Papua New Guinea, sex workers participating in an HIV prevention intervention reported gang-rape and harassment by the police as a serious barrier to their ability to practice safe sex.[30]

### Police harassment and possession of condoms as cause for arrest

In India, non-government organisations reported that sex workers were arrested even when they were not working as their identity was known to the police. Police harassment was listed as the sex workers’ primary concern. Physical abuse, demands of free and unprotected sex and manipulating the law to book sex workers was reported and carrying condoms was regularly taken as proof of their profession.[31] In 2011, a six country study of police practices in relation to sex workers commissioned by the Open Society Institute found; 80% of sex workers surveyed in South Africa reported they had been intimidated or harassed by police for being a sex worker or doing sex work, 85% of sex workers surveyed in Zimbabwe said they had been extorted by police, 80% of sex workers surveyed in Russia said police had confiscated their condoms, 50% of sex workers surveyed in Zambia said police destroyed their condoms and 75% of those who then worked did so without a condom, 60% of sex workers surveyed in Russia said police had used condoms as evidence of sex work, 52% of sex workers surveyed in the USA said that sometimes they did not carry condoms for fear of trouble from the police, 50% of outreach workers surveyed in Kenya said that police had harassed them during the course of their work. [32]

### Use of police crackdowns

In many settings police stage periodic crackdowns on sex workers to appease local residents, businesses or the political election agenda of a ruling party. Evidence has shown that law enforcement “crackdowns” only displace street sex work to other areas, sometimes for a limited time only.[33]. This displacement can divert sex workers from areas with established services to more isolated or dangerous areas. The US State Department noted in its 2002 report on human rights practices in Cambodia that the general police crackdown on sex work made sex workers even more vulnerable to intimidation, violence, theft, rape, and disease.[34] Crackdowns were particularly punitive in Cambodia following the introduction of the 2008 Law on Suppression of Human Trafficking and Sexual Exploitation. In some cases HIV positive sex workers were denied access to ARV and other medication while being detained.[35]

Evidence has shown that police raid and rescue actions often result in repeat trafficking or in increased debt for the sex worker if the rescued worker is bailed out by the brothel owner.[36] This requires sex workers to take on more clients to manage this debt. In India, a 2003 National Human Rights Commission (NHRC) report shows a significant number of repeat trafficking episodes: among rescued women, 17.5 percent had been to rescue homes once before, 1.8 percent twice before, and 6.6 percent more than twice before.[37] Many returned to the brothels from which they had been rescued to continue working. The report stated that this approach raises concerns related to state corruption, including bribes, extortion, sexual coercion, and complicity on the part of the police.[38] Moreover, rescues can be traumatizing, and girls face the risk of abuse in the safe house and can be re-trafficked.[39]

Shannon et al have described the effects of police presence on health and harm reduction services aimed at street-based sex workers in Vancouver, Canada.[40][41] Their 2008 and 2009 studies investigated female sex workers in Vancouver and found that a heavy police presence and crackdowns could displace populations away from health services. The displacement of sex workers to side streets and industrial settings presents a barrier to the success of health and support services aimed at addressing the HIV epidemic. Police presence was frequently associated with an increase in drug-related harms and a decrease in access to harm reduction services such as needle syringe programs. The authors suggested “enforcement based drug policies” facilitated drug-related HIV risk and that prohibitive sex work policies also facilitated HIV risk through sexual infection.

The harassment and arrest of peer educators and outreach workers by police, documented in India, Nepal and the Philippines severely compromises their ability to serve their constituency and provide condoms and safe sex information.[42] Incarceration and detention due to police crackdowns and raid and rescue attempts can result in criminal records and make sex workers vulnerable to sexual assault and coercion.[43]

### Implications for sex worker health and wellbeing and access to HIV prevention, treatment, care and support

Police harassment, violence and extortion have very real and serious consequences for the health and well being of sex workers. Violence is associated with unprotected sex and heightened risk of STI and HIV transmission.[44] In many settings police do not use condoms in coerced sex or rape. Paying bribes to police in order to avoid arrest means there is less money to pay for condoms and sex workers must accept more clients to make up for lost income. Not carrying condoms for fear of arrest has obvious implications for STI and HIV transmission. A person who has been a victim of abuse or violence can be stigmatised, or can cause fear in others.[45] This stigma can be a barrier to accessing health services, including treatment of injuries, and HIV/STI prevention services and treatment.[46] Marginalised or stigmatised people can be seen as deserving of violence and this can be internalised as guilt or self-blame. Living in constant fear of violence can harm a person’s self-esteem, and compromise their safe sex negotiation skills thereby their ability to protect themselves from HIV.[47]

### Positive examples of police practice and partnerships between police and sex workers

Despite criminalised environments, there are emerging examples of both sex worker programs working with police to create greater understanding of the sex industry within police ranks, to ensure better access to services and greater wellbeing for sex workers. In addition, there are also examples of police led interventions as well as reform measures taken by police institutions that are beginning to result in both better outcomes for sex workers and police. A selection of these programs are described in order to draw out the critical factors and key ingredients in changing the enabling environment in which sex work takes place to ensure that HIV prevention, individual and public health as well as HIV prevention and the promotion of human rights are the number one priority.

### Preventing violence towards sex workers

Resourcing Health and Education in the Sex Industry (RhED) is a service for the sex industry in Victoria, Australia. RhED uses a social model of health that incorporates harm minimisation, health promotion, social inclusion and community participation approaches to promote physical, emotional and social health and wellbeing for sex workers.[48] RhED’s Ugly Mugs project liaises with local police to report and prosecute perpetrators of violence against sex workers. The ‘Ugly Mugs’ Program aims to alert sex workers to dangerous individuals and situations in order to prevent further violence and harassment. It provides a non-judgmental and supportive reporting system for sex workers and an avenue through which sex workers can report people (Mug) who commit offences against them. Descriptions of Ugly Mugs and the incidents that took place are compiled into ‘Ugly Mug Reports’ which are circulated throughout the local sex worker and broader community. Reports are circulated by way of outreach and postings at RhED, brothels and community agencies, and relayed to the local state police, where they attempt to follow up the incidents using information and descriptions collected via the Ugly Mug reports. The Ugly Mugs Program was the recipient of the Australian Violence Prevention Award in 1996 and has been replicated successfully in other states and countries.[49]

### Police internships with organisations working with sex workers

Thailand’s Sex Workers in Network Group (SWING) provides services for male sex workers in Bangkok and has an intern program for police recruits that aims to build relationships of mutual respect to enable sex workers to access STI and HIV prevention and treatment without fear of arrest. Police cadets’ internship gives them first-hand knowledge of the working lives of sex workers. The police cadets study English and take part in other SWING program activities such as outreach. The program aims to sensitise future police officers to the needs of sex workers to foster supportive environments for sex workers and to create a cadre of police officers within the Bangkok police ranks that share an understanding and empathy for sex worker’s rights.[50]

### Police training and education

Save the Children’s Poro Sapot program works with police liaison officers and conducts peer education amongst police in various sites in Papua New Guinea (PNG]. The project, which operates in four cities across three provinces, is supported by Police Liaison Officers. The emphasis is on respect for and protection of the dignity of sex workers and peer educators as well as for gatekeepers and police. The aim is to create safe and HIV-protective environments within which social and commercial interactions can take place. This has resulted in HIV information and supplies now being actively requested by gatekeepers rather than being grudgingly received.[51]

### Addressing police harassment and violence through ongoing communication and joint workshops

In response to constant police harassment, the Vikas Jyot Trust (VJT) of India decided to target local police stations to talk about HIV awareness with operational police. Informal meetings with sex workers and police were held which proved to be a beneficial way to communicate issues of concern for sex workers. The police constables were sensitized to the problems facing sex workers and devised steps to lessen the harassment. Peer educators were introduced to police so they recognized them as a part of the program. Police constables are now informing VJT as soon as any sex workers have been arrested.[52] The Gates Foundation supported larger scale efforts at reducing police violence towards sex workers in Southern India with a multilayered program that strengthened sex worker networks and partnered the networks with legal and paralegal representation and also prioritised sensitisation training with police. The combination of efforts led to a significant reduction in sex worker experience of police harassment and violence.[53]

Keeping Alive Societies Hope (KASH) has worked with sex workers for over 7 years in the Nyanza province of Kenya. In Kisumu, Nyanza’s largest city, 60% of sex workers are HIV positive. Police violence has been widely observed and documented as a determinant of HIV risk. In 2007, KASH initiated joint workshops for police and sex workers to discuss, share and learn about HIV and AIDS and Kenyan laws on sex work. KASH selected 10 police officers and 10 sex workers as peer educators. This core group developed a data collection system to document abuses against sex workers, held regular workshops for their peers, and reconvened bimonthly to discuss their findings and strategize on how to address emerging patterns of abuse. The programme is now supported by the provincial police administration who have made it an integral part of all police training programmes in Kisumu.[54][55] Similar work is being supported by UNFPA in Ghana where female sex worker friendly police personnel are trained to provide protection to sex workers where appropriate. Regular meetings between sex workers and police representatives and training in human rights are designed to reduce harassment and discrimination by police.[56]

### Developing police HIV/AIDS instruction, strategy and protocols

The development of police strategy, instruction and standard operating protocols has been shown to have some impact in addressing HIV risk among key affected populations, including sex workers. In 2005 the Nepal Police released their HIV/AIDS Strategy and Work Plan that outlined the commitment of the Nepal Police to HIV prevention both among uniformed officers and the general population of Nepal including sex workers and other key affected populations. The document outlines the importance of collaborative partnerships between the police, public health and NGOs working with vulnerable groups. It also mentions the need for HIV training and sensitisation of high ranking police officers so that policy can translate to operational guidelines and practice on the ground.

Specifically in relation to vulnerable groups, the overall objective of the Nepal police is to ensure the creation of an enabling environment for supportive behaviour with vulnerable groups. In addition to regular sensitisation meetings, internships and field visits, the HIV/AIDS Strategy of the Nepal Police also includes a joint monitoring mechanism between the police, civil societies and vulnerable groups to monitor the behaviour of the Nepal Police. [57]

Further supporting reforms from the Nepal Police included the establishment of a Human Rights Unit in 2003 in order to promote and protect human rights in Nepal by implementing a range of human rights promotion measures into the operations of the Nepal Police. The Human Rights Unit is represented in all levels of the Nepal Police to train police on relevant human right issues, to monitor Nepal Police in the maintaining of human rights standards and to investigate cases brought to their attention by individuals or members of a community or network.

## Discussion

To the knowledge of the authors there are no rigourous peer reviewed studies that have examined the efficiency of police/sex workers collaborations or police led interventions or reforms on HIV risk behaviour or access to service. As a result there are limitations regarding the quality and quantity of data included in this review. While these programs are not extensively documented, a realistic review makes it possible to draw out common themes and key ingredients that can help foster better partnerships between police, sex workers and the programs that provide services to sex workers. It also highlights where research to further investigate this field is warranted. In promoting an environment where collaborations between police and sex workers can support enhanced HIV service provision it is clear that the following areas need to be considered.

### Leadership

While political leadership at a national and international level is critical, many of the examples described have resulted from local level collaborations between police and sex worker groups. Inherent is the need to identify champions from both the policing and the civil society, community-based and non-government organisations. The pursuit of collaborative leadership efforts can bring a sense of ownership, responsibility and support for partnerships. The Australian Government’s former aid agency, AusAID, supported senior police officials from Vietnam and Cambodia to Australia to partake in a three-week training program designed to develop and promote police leadership in responding to HIV in Asia. Further efforts to develop police leadership include the formation of the International Police Advisory Group (IPAG) under the secretariat of Law Enforcement and HIV Network. An IPAG delegation recently presented at the 2013 UN Commission on Crime Prevention and Criminal Justice and launched an International Statement of Police in Support of Harm Reduction and other evidence-based HIV Prevention Strategies for Key Affected Populations. [58]

### Civil society strengthening

It is clear that as civil society organisations, NGOs and community based organisations and networks gain strength, they are more able to represent the needs of the people they work with and more able to engage in discussions and collaborative program design with police. The ongoing support for these organisations is critical as is their need to articulate and develop advocacy and program design strategies that describe and outline how they will engage and work towards partnerships with the police. This paper briefly described the positive impact of these efforts in India and Kenya.

### Police reform in the context of HIV

Across regions with concentrated epidemics of HIV among sex workers there exist numerous reviews of the legal and policy environment in which sex work takes place and the changes in legislation that are required. This paper highlights that without an equal examination of the culture and practice of law enforcement, addressing the legal and policy environment is self-defeating.[59] Partnerships between police and sex workers are extremely difficult without some degree of police reform. While violence, arbitrary arrest and intimidation are the norm; partnerships with sex workers will remain unlikely.

The example of the Nepal Police in designing and implementing their HIV/AIDS Strategy effectively outlines a best practice response of police when working with key affected populations including sex workers. While acknowledging the need for further legal reform, resources and high level political will, the Human Rights Cell of the Nepal Police has been an important platform and link between civil society groups and the Nepal Police. Police reform in the context of responding to HIV among sex workers is being seen through the efforts of the Ukrainian Police Training Institute [60] and the ongoing efforts in the Kyrgyzstan Police Academy to update their police HIV training curriculum.[61] There is a need for the HIV community to identify broader police reform and professionalization opportunities that are often part of broader international development efforts and find a way to ensure that the HIV is included in these efforts.

## Conclusion

Police partnerships are not a substitute for law reform and should not preclude advocating for a more human rights based legal framework for the sex industry. However given that criminalization of sex work and sex work activities persists in a majority of countries in the world, creating police partnerships is a pragmatic option in support of the health and wellbeing of sex workers. The authors acknowledge that the examples of the programs described here warrant a more in depth analysis into the essential elements of HIV prevention programs that work with police in order to improve the health, wellbeing and quality of life of sex workers. Documenting these best practice examples and promoting further analysis begins to build an evidence base for the design of programs that can redress the barriers that law enforcement create and help contribute to greater access to services [especially HIV and STI prevention] and quality of life for sex workers and freedom from repeated incarceration and violence. Unfortunately these examples remain the exception rather than the rule.

At the intersection of policing and sex work and in pursuit of a more enabling environment for the provision of HIV services for sex workers it is clear that interventions need to be multi-layered, multi-sector and designed so that they can be scientifically evaluated. While these intervention and study designs are more complex, the research community and the institutes that fund HIV prevention research need to move beyond single interventions and prioritise the design of multi-faceted structural interventions that require a greater level of complexity and indeed a greater level of community and government involvement. This is really the last frontier of HIV intervention science: the science of the enabling environment. The very process of this type of research will lead to program designs that if enacted will contribute to the scale up of access to HIV prevention, treatment, care and support for sex workers and other key affected populations around the globe. In addition, it will also lead to the development of more humane and professional police services.
